# Focal Vibration Training (Equistasi^®^) to Improve Posture Stability. A Retrospective Study in Parkinson’s Disease

**DOI:** 10.3390/s19092101

**Published:** 2019-05-07

**Authors:** Francesco Serio, Cosimo Minosa, Matteo De Luca, Pierguido Conte, Giovanni Albani, Antonella Peppe

**Affiliations:** 1 Department of Rehabilitation, ASL Taranto, 74121 Taranto, Italy; cosimo.minosa@asl.taranto.it (C.M.); deluca_matteo@libero.it (M.D.L.); pierguido.conte@asl.taranto.it (P.C.); 2 Hospital Clinic “Le Terrazze” di Cunardo, Varese, 210365, Italy, 5 Department of Neurology and Neurorehabilitation, Clinical and Research Institute, Piancavallo, 28921 Verbania, Italy; g.albani@auxologico.it; 3 Fondation S. Lucia IRCSS, 00142 Rome, Italy; a.peppe@hsantalucia.it

**Keywords:** Parkinson’s disease, rehabilitation, focal vibrations, Equistasi^®^, falls

## Abstract

Background: For people with Parkinson’s disease (PD), falls are a critical point. Focal vibration training (FVT) may represent a valid tool to improve postural performances and reduce the risk of falls. The aim of this study was to evaluate the efficacy of FVT to improve the postural stability in PD patients. Methods: Since October 2015, 55 consecutive PD patients have been selected (T0) for an approach including FVT associated with a rehabilitative protocol (RP); after eight weeks (T1), those patients showing a relevant improvement in the clinical rating scales ((Timed Up and Go (TUG), Tinetti, Unified Parkinson’s disease rating scale (UPDRS) Part III, Berg Balance scale (BBS) and falls rate scale), continued with the FVT protocol (FVTRP group). The remaining patients continued with only the RP (RP group). In July 2018, we have extrapolated the data of the last clinical visit (T2) to observe any differences in the rate of falls. Results: The FVTRP group shows a decrement in the rate of falls from 2.1 to 1.25 (p 0.036) and a stability of the levodopa equivalent daily dosage (LEDD). The RP group shows an increment of LEDD and stability in falls. Conclusions: FVT has been confirmed as a valid tool to enhance the effect of the rehabilitation protocol aimed at postural training.

## 1. Introduction

Parkinson’s Disease (PD) is a neurodegenerative disease characterized by the loss of dopaminergic neurons, especially in the basal ganglia that leads to a progressive alteration of automatic movements such as gait and postural stability [1]. In addition to the pharmacological and surgical management, there are also complementary therapies [2], physical exercises and physical therapy, which have been actively studied in both long- and short-term periods [3,4]. Ultimately, it has been noticed that by focusing on proprioceptive exercises for posture instability, PD patients may receive a positive effect on axial symptoms [5,6] and on the number of falls [7,8], which not only represent a clinical critical point for people with PD, but even a cause of increasing costs for the health system [9,10]. Clinical and scientific reports support the knowledge that pharmacological and surgical therapies may impact only partially on postural performances [11,12,13,14]. On the other hand, a rehabilitative program focused on proprioceptive stimulation may strengthen the effects of therapy [6]. A two-month focal vibration training (FVT) resulted in efficiently reducing the rate of falls in people with PD [15]. Thus, it would be interesting to evaluate these effects for a longer follow-up. FVT is selectively used in the neurological rehabilitation [15,16,17], because of its effect on spinal excitability [18]. The tendon vibration may even induce an excitatory effect on the motor cortex, as suggested by the increased amplitude of motor evoked potential observed by transcranial magnetic stimulation applied over the motor cortex after muscle vibration [19]. Equistasi^®^ is a medical device registered by the Italian Ministry of Health (class 1, Ministry Code 342577 del 05/08/2010) and registered as a member of the National Assistance List, with the Trade Brand CE and authorized from 2010 for marketing. It is formed by a rectangular plate of 10 × 20 × 0.5 mm and with a weight of 0.17 g. It is exclusively composed of nanotechnological particles that transform the body temperature into a mechanical energetic vibration (0.8 N, 9000 Hz) [18]. This generates a movement of the muscle fibers of 0.02 mm, within the safety limit (0.12 mm) for damage of the human muscles [20]. The aim of this study is to evaluate retrospectively the effects of long-term FVT on postural performance of people with PD. 

## 2. Materials and Methods

### 2.1. Study Procedure, Observed Measures and Requirements for Obtaining the FV Equistasi^®^

This is a retrospective non-pharmacological observational study, in which 55 cases of PD patients (see Table 1) accessing the Department of Rehabilitation of Taranto ASL (Italy), from October 2015 to July 2017, were evaluated. We received authorization from the Ethics Committee with no. 184/18, and all patients gave their consent for using their own data. 

We used the Movement Disorders Society Unified Parkinson’s disease rating scale (MDSUPDRS) Part III (Motor Examination) [21], the Tinetti scale (Balance Evaluation) [22], Berg Balance scale (BBS) [23], the Timed Up and Go (TUG) [24] and the mean falls per month (FALLS). 

**Figure 1:**
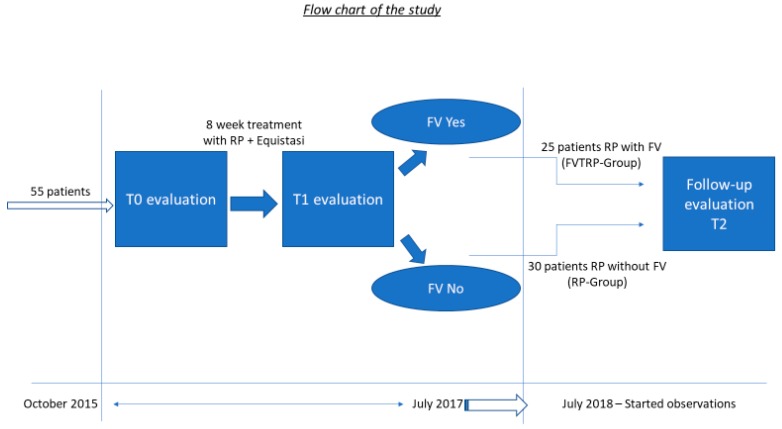
Flow chart.

The first part of the protocol (Figure 1) included an evaluation of clinical parameters at the first visit (T0) and after eight weeks (T1) of using three Equistasi^®^ devices, with two placed on the triceps surae muscle and one on the 7th cervical [15], for 2 h in the morning and 2 h in the afternoon for five days a week for eight weeks. We predefined that at the end of the eight weeks (T1), those patients showing an improvement of all four clinical measures greater than 20% were selected for FVT. The cut-off of 20% was established on the basis of the previous study [15], where, an improvement of 16% was significant (p < 0.01).

**Figure 2:**
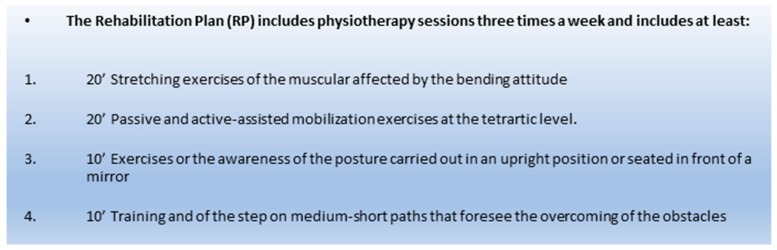
Rehabilitative plan (RP).

Those 25 PD patients obtaining the minimum improvement of 20% were admitted to receive the Equistasi^®^ devices completely covered in the cost by the National Health Service and continue their RP (Figure 2) with the addition of the FVT (FVTRP group). The protocol, in these patients, was three physiotherapy sessions per week associated with the Equistasi^®^ treatment for 2 h in the morning and 2 h in the afternoon for five days a week for at least one year.

Those 30 PD patients non-responding sufficiently to the Equistasi^®^ device, continued their RP without FV (RP group). 

After one year of treatment, all 55 patients were again evaluated (T2).

### 2.2. Statistical Analysis

The continuous variables were all evaluated with the Shapiro-Wilk test to verify their distribution of normality. For the evaluation between the groups, the Test Student was used for independent samples on the continuous variables while the Chi-squared test was used for the qualitative variables. For the evaluation between T0 and T2, repeated-measures ANOVA or the non-parametric test of Wilcoxon was used for the coupled sample if there was no distribution of normality (FALLS). In this case, we calculated the intervals of confidence of significance [25] by the Monte Carlo Method (MC) [26]. This offers the possibility to verify the adequacy of the estimate of the asymptotic P-value. The IBM Software SPSS V.22 with α = 0.05 was used and every test was evaluated with a two-sided method.

## 3. Results

Of the 55 patients that began this program, one patient (FVTRP group) died of myocardial infarction and one patient (RP group) dropped out because of hospitalization for an acute heart attack.

The two FVTRP and RP groups generated by the eight-week protocol, show homogeneous clinical variables (Table 2).

The mean time of the follow-up (from T1 to T2) was not significantly different between groups: FVTRP 20.5 months (SD 4.2), RP 21.15 months (SD 5.5) p = 0.633. 

At the end, in T2, the mean frequency of falls reduced significantly in the FVTRP group, while the RP group did not show any improvement (Table 3). 

The fallers in the FVTRP group in T0 were nine (37.5%) while in RP they were 16 (55.2%). In the FVTRP group the LEDD remained stable, while in the RP group it was significantly higher (Table 3).

## 4. Discussion

Muscle spindles are extremely sensitive to externally applied vibrations and, under such circumstances, they convey proprioceptive inflows to the central nervous system that modulates the spinal reflexes excitability, as well as the posture or the muscles responses elicited by postural perturbations. Vibrations may influence motor control: It has been proved that the vibration of axial muscles may produce systematic changes in the standing posture [27]. Vibrations may also influence human walking: In quiet standing, the tibialis anterior elicited a prominent forward body tilt, whereas vibrations of the hamstring and triceps surae eliciteda backward trunk; in the treadmill locomotion, hamstring vibrations produce a forward stepping [28]. Vibratory stimulation on trunk muscles has been used for therapeutic purposes in PD patients, providing an improvement of the trunk sway [29]. Even spatiotemporal parameters of gait in PD patients ameliorate after vibration and in particular in the stride length and cadence, after the erector spine muscle vibration [30]. FVT by Equistasi^®^ has been used, in a double-blind controlled study, in association with a physiotherapy program for training balance in PD patients. Forty patients with PD were randomly divided in two groups wearing an active or inactive device. All patients received a two-month intensive program of balance training. Both groups improved at the end of the rehabilitation period, but the Equistasi^®^ group showed a significant improvement at T1 on clinical scales with a sustained improvement at T2. Moreover, the Equistasi^®^ group showed a significant and sustained reduction of the falls rate, and, by a posturographic exam, the sway area and the displacement among the anteroposterior axis, improved only during the closed eyes test, when the degree of instability is higher [15]. 

FVT has achieved interesting results even with gait in PD patients: Ten patients after four weeks of FVT, showed an improvement of spatiotemporal parameters by a decrement of cadence, an increment of stride length, pelvis and trunk rotation and an increment of motion of the knee [31].

One of the limitations of this study is that we do not have available data of all clinical scores in T2, but only the falls rate and LEDD.

The present study confirms the clinical significance of FVT on postural performance in PD patients. In fact, the FVTRP group shows a significant reduction of falls at follow-up. Furthermore, in this group we also observed at the end of the study stability in the LEDD, even indicating a general improvement of motor status, over posture.

This opens new scenery for both management of the disease and the rehabilitative programs of these patients. 

Finally, to our knowledge, this is the longest follow-up study about FVT in PD patients, and the absence of reports of adverse effects, support the safety and tolerability of this approach, over the potential benefits. 

## Figures and Tables

**Table 1:** Characteristics of patients in T0 (n = 55); PD = Parkinson’s disease; y = years; LEDD = levodopa equivalent daily dose; s = seconds.

Characteristics of Patients	Mean (± sd)
Male/Female	37/18
Age (y)	69.33 (8.21)
Disease Duration (y)	8.09 (5.00)
LLD (mmg/die)	490.3 (222.4)
H/Y	2.58 (0.86)
UPDRS III	39.2 (17.9)
TINETTI SCALE	18.33 (6.2)
BBS SCALE	46.7 (6.2)
T.U.G. (s)	14.82 (5.1)

**Table 2:** Comparison to T0 between the focal vibrations training rehabilitative protocol (FVTRP) and rehabilitative protocol (RP) groups; we used T Student Test for independent samples.

	FVTRP (± sd) n = 24	RP (± sd) n = 29	p Value
Age (y)	68.6 (8.6)	69.4 (7.8)	0.773
Disease Duration (Y)	7.88 (5.6)	7.16 (4.4)	0.581
H/Y	2.73 (0.7)	2.55 (0.9)	0.761
LLD (mmg/die)	485 (176)	527 (228)	0.544
UPDRS III	37.3 (19)	39.4 (18)	0.671
Tinetti	17.3 (5.3)	19.4 (6.9)	0.222
BBS	41.04 (9.3)	43.45 (10.6)	0.378
TUG	15.9 (5.4)	14.41 (5.7)	0.267

**Table 3:** Differences between FVTRP and RP at follow-up; we used Monte Carlo (MC) method for the mean falls per month (FALLS) and repeated measures ANOVA for the levodopa equivalent daily dosage (LEDD); ° upper limit of the confidence interval of the Monte Carlo method lower than 0.05.

	FVTRP (± sd) n = 24			RP (± sd) n = 29		
	T0	T2	p Value	T0	T2	p Value
FALLS	2.1 (0.7)	1.25 (0.6)	0.036°	1.9 (0.6)	1.94 (0.8)	0.420
LEDD (mmg/die)	505.2 (158)	465.7 (169.1)	0.163	531.2 (227)	582.2 (239)	0.040
